# Fabricating Silicon Resonators for Analysing Biological Samples

**DOI:** 10.3390/mi12121546

**Published:** 2021-12-12

**Authors:** Momoko Kumemura, Deniz Pekin, Vivek Anand Menon, Isabelle Van Seuningen, Dominique Collard, Mehmet Cagatay Tarhan

**Affiliations:** 1 Graduate School of Life Science and Systems Engineering, Kyushu Institute of Technology, 2-4 Hibikino, Wakamatsu-ku, Kitakyushu-shi, Fukuoka 808-0196, Japan; momo@life.kyutech.ac.jp; 2 LIMMS/CNRS-IIS, Institute of Industrial Science, The University of Tokyo, 4-6-1 Komaba, Meguro-ku, Tokyo 153-8505, Japan; pekin.deniz@smmil-e.com (D.P.); collard@iis.u-tokyo.ac.jp (D.C.); 3 CNRS/IIS/COL/Lille University, SMMiL-E Project, CNRS Délégation Nord-Pas de Calais et Picardie, 2 rue de Canonniers, CEDEX, 59046 Lille, France; 4 Univ. Lille, CNRS, Inserm, CHU Lille, UMR9020-U1277—CANTHER—Cancer Heterogeneity Plasticity and Resistance to Therapies, F-59000 Lille, France; isabelle.vanseuningen@inserm.fr; 5 Division of Mechanical Science and Technology, Gunma University, 1-5-1 Tenjin-cho, Kiryu-shi, Gunma 376-8515, Japan; vmenon@gunma-u.ac.jp; 6 Univ. Lille, CNRS, Centrale Lille, Junia, University Polytechnique Hauts-de-France, UMR 8520—IEMN, Institut d’Electronique de Microélectronique et de Nanotechnologie, F-59000 Lille, France

**Keywords:** silicon, microelectromechanical systems, resonators, fabrication, biological applications

## Abstract

The adaptability of microscale devices allows microtechnologies to be used for a wide range of applications. Biology and medicine are among those fields that, in recent decades, have applied microtechnologies to achieve new and improved functionality. However, despite their ability to achieve assay sensitivities that rival or exceed conventional standards, silicon-based microelectromechanical systems remain underutilised for biological and biomedical applications. Although microelectromechanical resonators and actuators do not always exhibit optimal performance in liquid due to electrical double layer formation and high damping, these issues have been solved with some innovative fabrication processes or alternative experimental approaches. This paper focuses on several examples of silicon-based resonating devices with a brief look at their fundamental sensing elements and key fabrication steps, as well as current and potential biological/biomedical applications.

## 1. Introduction

Microtechnologies provide undeniable advantages in handling biological samples for biomedical applications. These benefits arise not only due to device characteristic sizes matching those of the targeted biological samples ranging from hundreds of nanometres to hundreds of microns [1], but also from the ability to achieve high-resolution displacement and force sensing down to sub-nanometre and piconewton levels, respectively [2,3]. These complementary features enable the accurate manipulation of biological samples with high spatial and temporal resolution [4] and have been attracting increasing attention as a technological solution for biological applications in recent decades.

With this breadth of microsystem characteristic sizes, the variety of biological samples that can be analysed is equally broad, spanning from molecular/subcellular samples such as proteins [5], DNA [6,7], and viruses [8,9]; to bacteria [10,11] and mammalian cells [12,13]; and up to larger samples such as cell spheroids [14] and even small animals [15]. Consequently, this wide range of samples that can be analysed using different microtechnologies enables their use in various biological assays. While some techniques target only label-free detection [16,17] or mass profiling [18,19], some others characterise mechanical properties to link these properties with progression in disease conditions, such as cancers [20,21,22], malaria [23], and anaemia [24]. Similar use of mechanical forces can help with analysing changes in cell morphology, orientation, and proliferation rate at cell–cell and cell–matrix junctions [4], and also under external forces to observe cell mechanobiology [25] and/or mechanotransduction [26]. 

There have been several methods traditionally used for biological and biomedical analysis at the microscale such as atomic force microscopy [27], magnetic tweezers [28], optical tweezers [29], micropipette aspiration [30], and microplate stretchers [31]. These techniques allow users to directly probe molecules, DNA, cell components, and cells. More recently, microtechnology developments such as microfluidics and microelectromechanical systems (MEMS) have enabled both higher throughput and more sophisticated functionality than conventional methods. Lab-on-a-chip devices that combine several functionalities on a single chip have been adapted for a wide range of targets, such as molecule–nucleic acid interaction investigated with surface plasmon resonance (SPR) [32], high-throughput single cell analysis by deformability cytometry [33], and cellular enrichment by means of electrical [34], acoustic [35], and hydrodynamic components [36]. Microfabrication of polymer structures can also be used for mechanobiology, as demonstrated by the use of PDMS microposts [37]. Although polymers enable relatively simple lab-on-a-chip device fabrication, silicon micromachining allows more advanced functionality, intricate designs, and fine feature control even at nanometre scales due to well-developed and sophisticated fabrication techniques [38].

Silicon-based MEMS provide much more than just advanced microfabrication possibilities. Actuators and resonators, for example, show tremendous potential as their highly sensitive measurement capacity and dynamic characteristics allow them to be excellent tools for sensing applications [36]. Such silicon-based MEMS resonators can be used for monitoring biochemical reactions [39], label-free detection of biological molecules [22] down to sub-attogram mass resolutions [40], or the detection of cells [39] at low concentrations. These capabilities can also allow resonators to be used for diagnosing specific diseases [39]. Well-established microfabrication techniques enable not only the development of devices at the nanoscale but also the ability to integrate multiple functionalities in a single chip [39].

Despite all the aforementioned examples, MEMS’s capacity for biological applications is frequently overlooked. This might be partially due to the need for costly microfabrication tools that are usually reserved for more solid-state applications. Another possible reason is that mechanical and electrical systems do not always exhibit optimal performance in liquid due to the formation of electrical double layers and high mechanical damping. However, solving these known issues with innovative fabrication processes or alternative approaches reveals the true potential of silicon-based MEMS devices to provide high signal-to-noise ratios, sensitive measurements, stable and high-resolution mechanical stimulation, automatable handling, and multiplexed functionality, all of which are critical for practical use in biological and clinical applications requiring high performance. 

The purpose of this review article is to introduce MEMS resonators as tools for biological and biomedical applications. Although cantilever-based resonators have been used extensively with excellent sensitivity and characteristics, there are also other silicon-based MEMS resonators and actuators that show significant potential for biological and biomedical applications. With this review, we list some approaches allowing high performance in MEMS resonators when analysing biological samples. After explaining key fabrication steps to enable optimal MEMS performance when performing measurements on samples in liquid, we also survey the biological analysis and biomedical applications performed by MEMS resonators and actuators. As there have been excellent reviews focusing on detection with cantilever-based resonators [41] and various principles of detection [38], we pay more attention to more intricate designs and methods in addition to the variety of targeted biological samples and their practical scientific and medical applications.

## 2. MEMS Resonators and Actuators for Biological Measurements

The actuation and sensing elements of a device play an important role in determining its performance for specific applications. Two actuation modes are used: static and dynamic. The static mode uses differential surface stress arising from target bindings to achieve detection. The dynamic mode, on the other hand, relies on shifts in the resonance frequency due to changes in the mass or spring constant of the system. As the dynamic mode exhibits a higher potential for sensitive measurements, our main focus is on devices actuating during the measurements. 

There are two main properties that characterize the resonance behaviour in devices working in the dynamic mode: resonance frequency and quality factor (Q-factor). Changes in the mass and/or spring constant result in a change in the resonance frequency. Thus, real-time measurement of the resonance frequency is an effective means to monitor the changes in the system mass and/or spring constant. Q-factor, on the other hand, is an important performance measure that can be described as the rate at which the device loses energy per vibrational period [42]. In other words, Q-factor corresponds to the amount of energy lost by the resonator during operation due to interaction with its environment or to intrinsic defects. Q-factor is critical as it is related to the ability to successfully achieve sensing in an environment. Mechanical damping in liquid decreases the Q-factor, which results in much lower sensitivity in resonance-frequency-based measurements. To ameliorate the performance in liquid, researchers have taken different approaches such as fabricating a channel embedded in the cantilever to handle liquids while the device as a whole operates in a vacuum [43], keeping the actuating and sensing elements in air while partially accessing the liquid sample medium via an air–liquid interface [12], improving the system to work better in liquid [44], and simply performing actuation and sensing after drying the liquid on the structures [8].

### 2.1. Common Means of Actuation

Both intricate MEMS devices and simple cantilever structures can be excited or actuated with various techniques. These can be classified in 5 categories [44] as optical [45], thermal [14], electrical [46], magnetic [47], and acoustic. MEMS specifically allows for built-in actuation, although some of the actuation techniques—e.g., optical—can utilise external stimulation without any dedicated integrated structure for the actuation of the system. 

As this review focuses on silicon-based MEMS devices, we will briefly introduce the most common actuation methods used in MEMS devices.

*Electrostatic actuation* is based on the electrostatic attraction between two electrically biased electrodes. These electrodes can be parallel plates [11] or interdigitated combs [48]. Electrostatic actuators are among the most well-developed structures in MEMS. They are popular not only for their simple structure and low power consumption but also due to their fast response. On the other hand, they require a relatively large footprint, which may not be suitable for some applications. 

*Electrothermal actuation* is based on thermal expansion of the actuator elements. A current passing through a beam raises the temperature via Joule heating, which deforms the beam. There are three commonly used types of configurations: U-shaped [49], V-shaped [50], and Z-shaped actuators [51]. Relatively small thermal strain can enable the generation of an amplified output force producing large displacement in one specific direction [52]. Therefore, one of the benefits of this type of actuation is the ability to induce large output forces with relatively low input voltages. However, high working temperatures may prohibit their extensive use in biological applications. 

*Piezoelectric actuation* is based on the electric dipoles in a material having different angles under stress [52]. Piezoelectric actuators provide large force with an excellent operational frequency bandwidth. Their compact design allows better integration in limited space, but they suffer from relatively small displacement ranges [53]. 

*Electromagnetic actuation* is based on a force generated by a current flowing through a wire coil in presence of a magnetic field [53]. Despite the difficulty in manufacturing and relatively large dimensions, electromagnetic actuators provide quick and large displacements. They are easy to control and exhibit high precision.

### 2.2. Common Sensing Techniques

Various sensing techniques have been used based on the requirements of the targeted application. Optical techniques, such as laser Doppler vibrometry, are some of the most commonly used sensing mechanisms for cantilever-based detection. Image analysis can also be applied to displacement sensing to perform sensitive measurements using Moiré Fringes [34]. There are several other techniques that can be integrated directly with a MEMS device and implemented for sensing in resonating and actuating devices.

*Capacitive sensing* is a method providing high sensitivity based on the changes in capacitance between two electrodes similar to electrostatic actuators [7]. The response is fast, and the measurable bandwidth is large. However, the large dimensions required for accurate measurement are a limitation that can prohibit its use for certain applications.

*Piezoresistive sensing*, based on the piezoresistive effect, is one of the most commonly used methods for force measurements. The resistance value of a piezoresistive element changes when undergoing strain and deformation. This type of sensor features high bandwidth and large frequency response. Although these sensors are advantageous with relatively simple fabrication processes, sensitivity to ambient temperature is a limitation. Furthermore, unlike capacitive sensors, measurement requires a flowing current through the piezoresistive element, which results in relatively large power consumption.

*Piezoelectric sensing* uses materials that provide a direct transduction mechanism to convert mechanical signals to electrical signals and vice versa [54]. These sensors have wide measurement ranges and bandwidths. Although complex manufacturing requirements are a disadvantage, sub-µN resolution makes them an essential sensing mechanism at the micro-/nanoscale.

### 2.3. Biological and Biomedical Use

Resonating and actuating MEMS devices have been used for a variety of biological and biomedical applications by analysing biological molecules, subcellular components, cells, and multicellular samples. These biological samples are naturally found in liquid-based environments where resonators conventionally suffer from poor performance due to viscous losses. Thus, several methods have been developed to maximise the performance of MEMS resonators. One of the prominent approaches is to perform a dip-dry-measure cycle in which the sensor is first immersed in the sample liquid to allow target molecules to attach onto the probe. The sensor is then removed from the liquid and dried, allowing the measurement to proceed in either air or vacuum, and the resulting shift in the resonance frequency is identified. Another approach is to perform continuous measurement. To achieve continuous monitoring, measurement must be performed with the probe directly in contact with the sample liquid to allow interaction with the target particles. Manalis et al. developed an elegant implementation of this technique by designing a microfluidic channel fully contained inside a cantilever. Using this kind of suspended microchannel resonator, resonance measurements can be performed continuously with the sensor contained in vacuum while the samples can be handled by controlling the flow through the internal channel [11,55,56]. Other researchers have developed systems with the probe tip working at an air–liquid interface to access the samples without immersing the sensitive actuation and sensing elements in the liquid [57,58].

Although not covered in this review, MEMS actuators and resonators have been used as components in various other biomedical applications in addition to biomarker detection and direct sample characterisation. A good example is the use of MEMS mirrors for further miniaturisation of endoscopes [59] and optical coherence tomography [60]. Moreover, gyroscopes and accelerometers have been some of the key elements in consumer health electronics in the recent years. We limit this review to MEMS resonators used for analysing biological samples from molecules to whole organisms.

## 3. Fabricating MEMS Devices

The techniques involved in fabricating a device for analysing biological samples vary depending on the properties and abundance of the target sample. Samples demanding relatively large structures—e.g., deep channels and steep walls—can benefit from silicon-only devices while samples being small in size and/or having low concentration might require combining silicon with other materials—e.g., nitride and carbide—to improve the detection sensitivity. In short, the biological sample to analyse determines the development of a MEMS device at different levels, e.g., design, material, and fabrication process.

### 3.1. Common Device Structures

**Figure 1:**
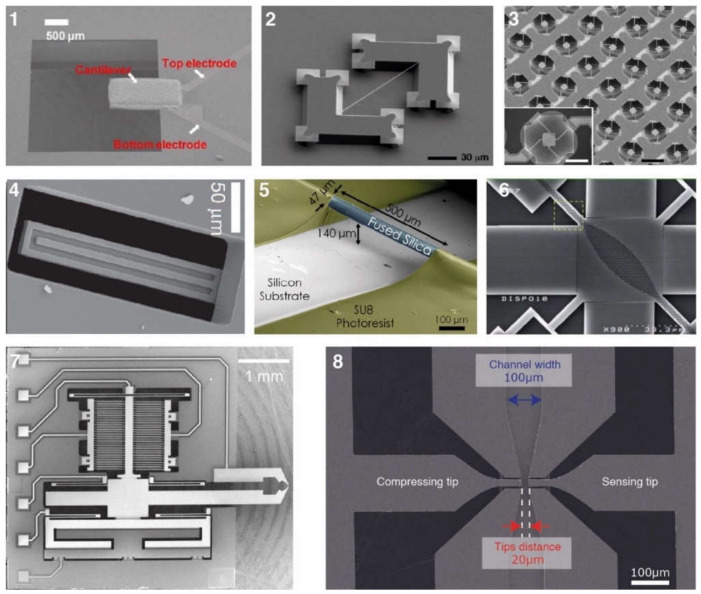
Examples of different types of silicon-based resonant MEMS applied at the subcellular level. 1–3 are suspended structures of (**1**) cantilever type (reproduced from [61], with the permission of AIP publishing), (**2**) bridging type [62] (Copyright Elsevier 2008), and (**3**) plate type [12] (with granted permission from PNAS). Structures 4 to 6 have integrated channels as in the cases of (**4**) cantilever type [11] (reprinted by permission from Springer Nature [11] Copyright 2007), (**5**) bridging type [63] (reproduced with a permission from ACS), and (**6**) plate type [64] (reproduced with permission from The Royal Society of Chemistry). MEMS squeezers include (**7**) microgrippers [57] (CC BY license) and (**8**) fluidics-integrated devices [65] (CC BY license).

There are various silicon-based resonating structures that have been used for analysing biological samples. The most common of them can be grouped in three design types: suspended structures, e.g., cantilever, bridge, or plate geometries; suspended channel devices, e.g., cantilever (also known as suspended microchannel resonator), bridge, and plate geometries; MEMS squeezers, e.g., microgrippers and fluidics-integrated devices (Figure 1). Here, we have a brief look at their common properties.

#### 3.1.1. Suspended Structures

*Cantilever beam structures* are some of the most commonly used geometries for detecting biological samples. The detection is based on changes in the surface stress (bending or static mode) or in the resonance frequency (dynamic mode) [66]. Attachment of a target sample on the suspended structure results in a decrease in the resonant frequency due to added mass. With a calibrated sensor, the shift in resonance frequency quantifies the mass of the captured sample. These resonators are frequently made of silicon, silicon nitride, and metals. Polymers, having mechanical properties with Young’s moduli lower than silicon, are available as alternative material candidates when higher sensitivity is required [67], e.g., at molecular and subcellular levels. 

The fabrication process of cantilever beams includes either or both surface and bulk micromachining techniques (see Section 3.2). Different shapes and sizes can be fabricated as single structures or arrays of large numbers of elements. Cantilevers are versatile devices allowing mechanical, optical, electrostatic, and electromagnetic means of actuation and sensing [68]. Fabrication steps depend on the selected actuation and sensing mechanisms. For example, some electrostatic methods require metal deposition [69] while piezo-resistive ones necessitate ion implantation [70]. 

*Bridging beam structures*, known as doubly clamped resonators, can achieve mass sensing at the attogram level [71]. Silicon nitride [71] or silicon carbon nitride [62,72] are examples of thin-film materials that are used in the fabrication due to their elastic properties. Bridging beam structure devices have been applied to detect proteins [62,72]. These devices used chemical vapour deposition (PECVD) for thin-film deposition, electron beam lithography for nanometric patterning, and wet etching (KOH) for the release process.

*Suspended plate structures*: The variation in the vibration amplitude along the length of a cantilever beam can be considered a limitation of such structures because the mass sensitivity is linearly proportional to the square of the vibration amplitude of the sensing beam [12]. As such, the same analyte can produce different signals depending on where it is positioned along the cantilever. A four beam–spring structure, however, can minimise the variation amplitude across the platform and decrease the detected mass variation to 4% [12]. The larger relative size of the attachment surface allows plate structures to be used for analysing not only proteins [73] but also whole cells [12,42,43,74,75]. Besides detecting mass for protein analysis [73], suspended plate devices have been used to detect cell mass in a continuous format over longer periods of time, thus allowing cell growth monitoring [12,42,74] and mechanical characterisation of cells [43,75]. Fundamental microfabrication techniques are used such as oxidation, deposition (e.g., Au, SiO_2_, SiN), and etching (dry, wet, and vapour).

Due to similar geometries, we can also mention the use of suspended membranes in this section. Although they are not supported by four beams, this type of sensing platform is used for the attachment of samples such as viruses [76]. The fabrication process includes chemical vapour deposition for SiN and sputtering for AlMo membranes, reactive ion etching of the patterned layers, and wet etching (KOH) of the silicon layer [77].

#### 3.1.2. Suspended Channel Structures

Cantilever beam structures have proved to be very sensitive when working in air and vacuum [66]. However, the damping effect of a surrounding liquid dramatically reduces the Q-factor, making the use of such devices less suitable for monitoring in liquid. To overcome this limitation, Manalis et al. embedded a microchannel within the suspended beam structure [11]. A sample solution continuously flows through the channel and delivers biomolecules, cells, or synthetic particles resulting in a total mass change inside the channel due to the difference between the mass of the analyte and that of the displaced fluid, i.e., buoyant mass. This change in mass is monitored via the change in the resonance frequency of the resonator [11]. Thus, the target samples inside the suspended channel can be analysed by characterizing the dynamic behaviour of the cantilever beam resonating in vacuum [78]. Suspended fluidic channels have been embedded in several resonating structures.

*Cantilever-type suspended channel structure:* The most commonly used suspended channel structure is in a cantilever beam format. One example of this device design featured a detection channel connected to two bypass channels embedded in a cantilever beam, which was driven electrostatically, while different biological samples passed through the channel. The vibration amplitude of the cantilever structure was monitored with a laser and a position-sensitive photodetector [11]. While electrostatic actuation is the most common method of actuation for cantilever-type suspended channel resonators, some devices have used an external piezoceramic actuator [39,40,79]. Similarly, in addition to optical sensing mechanisms [13,35,38,79,80,81,82], piezoresistive sensing can also be applied to achieve sensing [39,40,70]. Starting with detecting the buoyant mass of the target sample [11], these devices have demonstrated the measurement of cell density [81], volume [81], growth [80], deformability [35], and mass accumulation rates [38].

Early examples of suspended channel resonators were used to perform molecular analysis and, thus, had small channels [55]. Surface micromachining techniques were used to fabricate the channel with silicon nitride deposited by chemical vapour deposition (LPCVD). Using polysilicon as a sacrificial layer deposited between structural layers and subsequently removing it to free the cantilever was the key to forming the suspended structure. After these early demonstrations, a new channel fabrication process for cantilever-type devices was introduced. The process has two key fabrication steps: dry etching and wafer bonding [11]. Following a dry etching step to define the channel, two silicon wafers were bonded to form the embedded channel. Glass was used for vacuum sealing by bonding with the main silicon structure to enable optical sensing [78]. The silicon thickness and channel height, varying from sub-micrometre to tens of micrometres, depends on the dimensions of the target biological sample. Some applications required sensing at multiple positions, which resulted in the development of devices with channel-embedded cantilever structures connected in series [38,56]. Dense arrays of these structures lead to difficulty in aligning optical sensing elements. Therefore, such devices benefited from on-board piezoresistive sensing [39,40,70], which required doping the silicon wafer through ion implantation as an extra fabrication step in addition to the process developed for optical sensing.

*Suspended bridging channel resonators* are coupled at both sides of the channel. Examples of this geometry were used to demonstrate mass detection while allowing optical monitoring [83,84]. The optical analysis provided additional information, i.e., reflectivity [63]. To provide an optical view, the suspended bridging channels had to be transparent, at least on the observation side. This could be achieved by either forming the channel with transparent polymers, e.g., parylene [83,84], or by using silica microcapillaries [63].

*Suspended channel-in-plate resonators* benefit from achieving detection with less mass variation due to the device geometry, as explained previously [12]. Compared with cantilever or doubly clamped beams, suspended plate resonators generally exhibit higher Q-factors [64]. Although these devices have not yet been tested with various biological samples, several chemical solutions and biological buffers were tested with these devices [64,85]. Similar to the process of suspended channel resonators in a cantilever beam, fabrication steps include dry etching of silicon (partial RIE and DRIE) and wafer bonding (silicon–silicon and silicon–glass). 

#### 3.1.3. MEMS Squeezers

The suspended structures mentioned above are fabricated using fundamental micromachining techniques. However, the purely mass-based sensing techniques discussed thus far do not fully capitalise on the breadth of capabilities that MEMS have to offer. Integrating microfluidic channels with other MEMS elements can enable both mechanical and electrical stimulation of samples in a controlled manner. MEMS displacement and force sensors can achieve high resolution down to sub-nanometre and sub-nanonewton levels, respectively [1]; thus, a wide range of target samples can be handled and analysed: from DNA bundles [57] to aquatic microorganisms [47]. We can group these devices into two main categories: microgrippers, as tweezers to reach the target sample in a solution, and fluidics-integrated devices, which include fluidic features that transport the target samples to the actuating and/or sensing elements. 

*Microgrippers* have one or two tips to manipulate a target sample, which can be as small as a single microtubule [86] or as large as a fruit fly [15]. Due to the possibility of mechanical stimulation, analyses are performed using mechanical parameters, such as force [15,48,87], stiffness [57,58,87,88,89,90,91], and viscosity [57]. The demonstrated devices are fabricated with standard silicon micromachining techniques, which make electrostatic actuation and capacitive sensing easily accessible as no extra fabrication steps are needed. Some devices, on the other hand, integrate polymers, e.g., SU8, especially when the actuation is provided electrothermally [50,92]. 

Starting with a silicon-on-insulator (SOI) wafer, fabrication of MEMS elements primarily involves the dry etching of silicon (DRIE) to form device features and their subsequent release with wet (or vapour) etching of the buried oxide (BOX) layer. Microgrippers targeting molecules, e.g., DNA or microtubules, require another key step, i.e., anisotropic etching of the bulk silicon with potassium hydroxide (KOH) or tetramethylammonium hydroxide (TMAH). These solutions etch crystal planes in the silicon lattice at different rates and, thus, allow the formation of sharp tips [93] capable of handling such small samples. This etching process also necessitates the protection of some of the device sidewalls, therefore, additional steps of oxidation and chemical vapour deposition of silicon nitride are needed [7].

*Fluidics-integrated devices* have structures similar to microgrippers, but the tip features are positioned on either side of a channel or trapping site [34,65,94,95]. These devices have been used to analyse targets from collagen fibres [96] to cell spheroids [14]. Not all of the devices introduced in this section have resonating structures. However, we include such actuating devices due to the similarity of the designs, fabrication techniques, and target samples. Similar to microgrippers, the majority of these devices are fabricated using dry etching (DRIE) of the silicon and wet etching of the buried oxide layer to release the moveable structures. Some devices need silicon–glass bonding [34], while some others use PDMS to form a channel [97]. In addition, several devices were fabricated with the PolyMUMPs^TM^ process [95,98,99]. PolyMUMPs^TM^, the acronym for the polysilicon multiuser micromachining process, is a commercially available micromachining process. It provides a three-layer polysilicon surface, bulk micromachining process, two sacrificial layers, and one metal layer. This technique can be used to fabricate 3.5-µm tip structures suspended 2 µm above the silicon surface to manipulate cells in the channel [95].

### 3.2. Fundamental Fabrication Processes

Each device group mentioned in the previous section has many different resonator designs for analysing biological samples. However, there are many similarities in the key fabrication steps, as discussed in previous sections. This section gives a brief introduction to the essential common processes.

Fundamental micromachining processes can be grouped in two major categories: surface micromachining and bulk micromachining. Surface micromachining builds structures over the silicon surface by depositing layers. Depositing thin material layers and suspending them using sacrificial layers allow surface micromachining processes to perform well when used to fabricate suspended resonators, especially when high sensitivity is needed. As integrated circuit (IC) technology uses the same fabrication techniques, resonators built with surface micromachining can be integrated easily with IC components. Bulk micromachining, on the other hand, etches the silicon substrate itself to form structures. It allows building high-aspect-ratio structures including channels, chambers, and walls. That is why it has been a preferable approach for fabricating channel-integrated systems targeting relatively larger samples such as cells that require deeper features. Many MEMS devices benefit from both surface micromachining and bulk micromachining techniques.

Silicon has traditionally been the most commonly used material for MEMS devices. However, there are several other materials that are essential for improving device performance or for forming particular geometries. Silicon nitride (SiN), aluminium nitride (AlN), titanium nitride (TiN), and silicon carbide (SiC) are some of the most commonly used materials to fabricate thin beams for high-sensitivity resonators because of their mechanical, electrical, or thermal properties. Sacrificial layers, e.g., silicon dioxide (SiO_2_), polycrystalline silicon (polysilicon), and photoresists, are used during the fabrication of those thin beams. Besides these materials, MEMS devices often also use metals, e.g., gold, aluminium, chromium, nickel, and titanium, either as structural elements or as etching masks during the fabrication process. Polymers can also readily be integrated with MEMS devices according to the required chemical, mechanical, electrical, or thermal properties [63].

*Deposition*: One of the first steps of a standard process is deposition. Physical vapour deposition includes methods such as evaporation of a material (thermally or by an electron beam) and sputtering, which releases a target atom using energetic particles. These techniques are primarily used to deposit metals. Sputtering can also deposit SiO_2_, which can be grown directly on a silicon surface via thermal oxidation as well. Chemical vapour deposition (CVD) involves flowing precursor gases over a sample to react with the substrate and form a material layer. The resulting layer can be used as a structural or sacrificial layer. SiN, SiCN, AlN, and SiO_2_ can grow on silicon with this technique. The most commonly used CVD techniques for the devices covered in this review are low-pressure CVD (LPCVD), which functions at a reaction chamber pressure below 1 atm [57,100], and plasma-enhanced CVD (PECVD), which uses plasma to enhance deposition in a low-pressure chamber [12,70]. Polymers, e.g., parylene, can also be used among these processes, as in the example of building suspended channels to allow optical monitoring [63]. In addition, electroplating—using electrical current to coat metal on an electrode—is an alternative way to build relatively thicker electrodes, e.g., nickel [47].

*Patterning*: Deposited materials must be patterned to become functional elements. Among several available methods of patterning, photolithography is the most commonly applied technique. A photosensitive material, e.g., photoresist, changes its physical properties when exposed to light. Selective exposure, either with a mask or maskless (direct writing techniques), allows removal of the undesired resist areas. To build structures at the nanometre scale, electron-beam lithography is used. Changing the means of exposure from light to a beam of electrons mitigates resolution limitations due to the diffraction of light [101]. The pattern formed in the photosensitive material can then be transferred to the material below through a subsequent etching step.

*Etching*: There are two categories of etching processes: wet and dry. Wet etching uses chemical solutions to dissolve the material to be removed. Deposited materials can be etched selectively in specific solutions that do not damage photoresist, thereby allowing the photoresist to protect the areas underneath and form a desired pattern in the underlying material. Wet etching processes can be isotropic, with a uniform etching rate in all directions, or anisotropic, having different etching rates according to the crystal structure of the substrate. For example, the etching rate of silicon’s <100> plane is much faster (two orders of magnitude) when compared to its <111> plane in a KOH solution. This plane-specific etching results in 54.7° walls when a (100) silicon wafer is etched with a KOH solution. Dry etching uses reactive gasses in a plasma environment to remove material. Reactive-ion etching (RIE), a commonly used dry etching technique, provides anisotropic etching unless high plasma densities are applied. A process based on RIE, deep reactive-etching (DRIE), has become critical to obtain high-aspect-ratio structures or deep holes with vertical sidewalls. The Bosch process, the main technology for DRIE, uses two main elements: a very small isotropic etch followed by a passivation layer. Repeating these alternating steps, a silicon substrate can be etched for hundreds of micrometres with vertical walls. Another key etching process that can be considered dry etching is vapour etching. Achieving isotropic characteristics without using wet etching is critical to release suspended structures by removing sacrificial SiO_2_ layers. The use of a vapour-phase etchant prevents stiction, where a suspended feature is pulled down and immobilised on the substrate below through surface tension from a liquid etchant. Vapours of hydrogen fluoride (HF) and xenon difluoride (XeF_2_) are commonly used solutions for vapour etching [12,57].

Lift-off processes can be considered as an alternative to etching [101]. Instead of patterning and etching a deposited material, a sacrificial layer is instead used to selectively prevent adhesion of the deposited layer to the substrate. Unlike the etching process, a photoresist is first patterned directly on a substrate, which is followed by the thin-film deposition. When the sacrificial layer is removed, the desired structures remain. This process is beneficial when an underlying layer can be affected by the etchant or if the deposited material is difficult to etch. 

*Wafer bonding* is another key fabrication step used in suspended channel structures and some of the fluidics-integrated MEMS squeezers. The suspended channel devices use this technique for two different purposes. The first is the formation of channels embedded in a silicon structure. A partially etched silicon substrate is bonded to another silicon substrate to complete the channel. The etched areas correspond to the interior of the microchannel in which biological samples flow. The second purpose is bonding the silicon wafer with glass for hermetic sealing. As a result, the suspended structure can resonate in vacuum, providing very high sensitivity. Some of the fluidics-integrated MEMS squeezers require silicon–glass bonding to seal the channel for analysing biological samples. Various bonding techniques are used: plasma activated bonding [94], glass–silicone anodic bonding [11,14,34,102], glass frit bonding [103], and fusion bonding [79,80]. Polymer structures can also be used for similar channel-forming purposes. PDMS is a popular material to form channels or microwells [12,47,97,104] for handling biological samples.

MEMS devices usually require proper functionalisation of the structure surfaces prior to biological use. These are specific to the target samples, fabricated material, measurement technique, and analysis purposes. Functionalisation steps have been partially reviewed in other articles [68,105,106] and are not covered in this review.

A summary of the common device structures introduced in this section is provided in Table 1. Each device type is classified according to its typical sample targets, measurement parameters, and key fabrication steps.

## 4. Biological Applications

**Figure 2:**
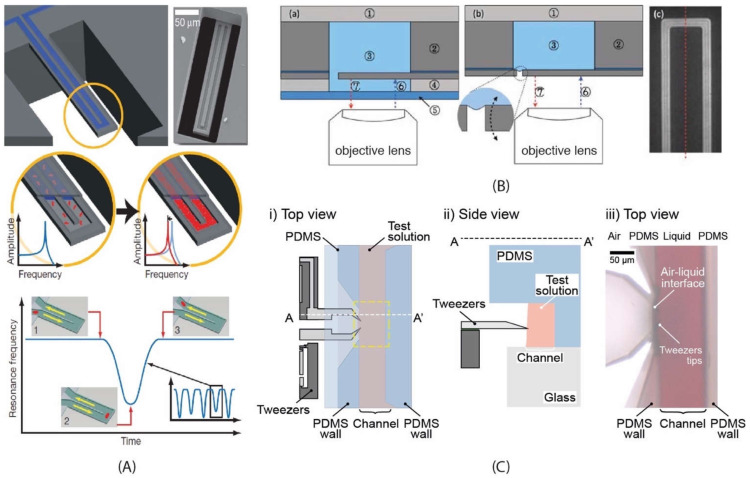
Examples of silicon-based resonant MEMS applied at the subcellular level. (**A**) Suspended channel resonator described by Burg et al. [11] (reprinted by permission from Springer Nature [11] Copyright 2007). (**Top-left**) the schematic representation of the resonator and SEM image of the cantilever (**Top-right**). The bottom side of the channel was etched open intentionally for visualizing the fluid conduit. Molecules flow continuously through the channel. Species that have the correct affinity bind to immobilised receptors on the channel walls and accumulate (**Middle panel**). In another measurement mode (**Lower panel**), particles flow through the cantilever without binding to the surface. The signal depends on the position of the particle inside the channel (numbers 1 to 3). The exact mass excess of a particle can be quantified by the peak frequency shift induced at the apex. (**B**) Schematic representation of the cantilever system used by Park et al. to improve the quality factor (50%) and signal-to-noise ratio (5.7-fold) by working at an air–liquid interface [112] (reproduced with permission from The Royal Society of Chemistry). They demonstrated the detection of insulin and monitored enzymatic activity between SOD1 and proteinase K [113]. Figure adapted [112] from with permission from The Royal Society of Chemistry. (**C**) Microgrippers, described by Tarhan et al., inserted only a very small area of their tips in a solution to perform titration experiments on a DNA bundle. The resonating and sensing MEMS elements working in air provide optimum MEMS performance [57,58,89]. (**i**) and (**ii**) are the schematic view (top and side) of the brightfield microscopy image showing tips of the microgripper access to the channel wall with a red solution (**iii**) (CC BY license).

As silicon-based MEMS resonators mature, the biological samples they analyse and biological applications they perform become more intricate. Burg et al. used suspended channel resonators to detect avidin and biotinylated-BSA [55] in one of their earliest demonstrations (Figure 2A). About a decade later, a similar device was used to assess drug sensitivity of single cancer cells by measuring mass accumulation rate [38]. Similarly, it took over a decade for microgrippers to progress from capturing their first DNA bundle [93] to constructing a chromatin analogue for testing the epigenetic effects of chemicals [90].

### 4.1. Working at the Molecular/Subcellular Level

#### 4.1.1. Targets

Quantitative detection of proteins, nucleic acids, exosomes, or viral particles is required in a wide range of activities such as drug dosing, clinical diagnostics, or protein characterisation. Both proteins and nucleic acids can be used as highly specific biomarkers for numerous diseases including cancer. Here, we discuss some typical biological samples that have been targeted by devices introduced in Section 3.1.

*Molecules and proteins* were analysed mainly with suspended structures and suspended channel structures except for fibrous targets, e.g., microtubules [86] and collagen fibres [96,100], which were handled by MEMS squeezers. This wide range of target samples includes mycotoxins, which are toxic chemical products produced by fungi. Ricciardi et al. presented the first successful immunodetection of low-concentration toxins (3 ng mL^−1^ for aflatoxins) using cantilever resonator arrays [109], which was later improved by two orders of magnitude (40 pg mL^−1^) by the same group [110].

Cantilever structures have also used to detect proteins. Tumour-homing peptides that target tumour vasculature are considered to be a promising agent for cancer detection at an early stage [111]. Puiggalí-Jou et al. used silicon-based resonators to demonstrate biorecognition between an engineered CREKA, a linear peptide that specifically binds to clotted-plasma proteins in tumour vessels, and clotted-plasma proteins (fibrin and fibrinogen). Although the minimum detection limit (100 ng mL^−1^) required further improvement to be practical in diagnostic settings, this was a notable attempt to comprehend biological interactions and their implications in the field [111]. 

Park et al., pursued a different approach to improve their detection limit. They tried to decrease the damping effect of the liquid around their cantilever structure by working at an air–liquid interface [112]. One side of the cantilever structure faced the microfluidic channel while the other side was facing air. The cantilever structure was surrounded by the rest of the silicon wafer with a small slit (6 µm) in between (Figure 2B). The high surface tension of the small meniscus formed around the cantilever kept the liquid in the channel. As a result, only half of the cantilever structure was in contact with the liquid, which improved the Q-factor (50%) and signal-to-noise ratio (5.7-fold). After an initial test detecting IgG antibodies [112], they used their device for direct detection of insulin (0.4 ng mL^−1^) [113]. The same device also demonstrated continuous monitoring of proteinase K—superoxide dismutase 1 (SOD1) enzymatic reactions [113].

Other groups have also used their resonators to detect antigens and antibodies. Gupta et al. developed their biosensor design by detecting BSA and IgG [107]. Their group, Bashir et al., later showed excellent demonstrations of suspended plate resonators. Zheng et al., on the other hand, detected BSA and IgG to demonstrate the use of diazonium-salt-induced surface modification as linker chemistry for the biofunctionalisation of glassy nanostring resonators [72]. 

Unlike previously mentioned studies, Brunetti et al. targeted antigens and antibodies for medical purposes to develop a rapid analysis technique for malaria vaccine candidates [117]. Their mechanical assay provided a direct, one-step, label-free quantitative immunoassay with a detection limit of a few pg mL^−1^ (or sub-pM concentrations) reaching the level of the conventional, multistep, enzyme-linked immunosorbent assay (ELISA) used currently in the field. 

Prostate specific antigen (PSA) is an important protein target as it is associated with cancer. Waggoner et al., used suspended plate resonators to achieve a relatively uniform frequency response for the bound protein mass regardless of its position on the sensor [73]. They reached detection concentration thresholds of 50 fg mL^−1^ (or 1.5 fM). Another demonstration for cancer diagnosis was provided by Choi et al. They targeted matrix metalloproteinase (MMP) in blood droplets of lung cancer patients using a cantilever biosensor with a detection sensitivity of 0.05 nM and correlated the secretion level of MMP2 molecules and the level of cancer metastasis [118]. Suspended channel resonators have also been demonstrated for detecting cancer biomarker molecules. Von Muhlen et al. used suspended channel resonators to detect activated leukocyte cell adhesion molecule (ALCAM) in undiluted serum with a detection limit of 10 ng mL^−1^ [115].

*Nucleic acids* were some of the earliest detection demonstrations using cantilever structures including double-stranded DNA [101] and single-stranded DNA [17]. Later came the demonstration of DNA hybridisation [61,119], digestion [120], and rheological characterisation [114], all with cantilever structures. Using suspended channel resonators, Olcum et al. weighed self-assembled DNA nanoparticle structures. They reported measuring 0.85 attograms, approaching the thermomechanical noise limit and enabling precise quantification of particles down to 10 nm [79]. A more recent study detected a cancer-associated miRNA expression profile from cell lysates and another one associated with hepatocytes derived from necrotic liver tissue [121].

Another resonant MEMS strategy targeting DNA was described by Tarhan et al. They used a microgripper to capture a DNA bundle in a platform to perform real-time, label-free, and substrate-free mechanical characterisation [57]. By integrating the microgripper with a microfluidic channel, titration experiments were performed on a DNA bundle (Figure 2C). Changes in the DNA bundles were monitored by continuously measuring their stiffness and viscous losses as a result of changes in solution pH (2.1 to 4.8); different cation concentrations, i.e., Ag^+^, Na^+^, K^+^, Ca^2+^, Mg^2+^, Zn^2+^; and the introduction of other molecules [57,58,89]. 

*Viruses* have thus far been detected primarily by cantilever structures. Gupta et al. were able to detect a single virion of vaccinia virus and determine its mass as 9.5 fg [8]. Later, Johnson et al. [108] used cantilever beams driven by thermal noise and a PZT piezoelectric ceramic as resonating sensors to measure the mass of the same viral species. Two sizes of cantilever were used. The average mass of a vaccinia virus particle was measured to be 12.4 ± 1.3 fg using cantilevers of 21 µm × 9 µm size and 7.9 ± 4.6 fg with cantilevers of 6 µm × 4 µm size. Ilic et al. also investigated the effect of the cantilever size on sensitivity for the detection of baculovirus particles [9]. They established that as the length of the cantilever decreased, the sensitivity increased. Cantilevers of 0.5 µm × 6 µm were able to show mass sensitivities around 10^−19^ g Hz^−1^ corresponding to a mass of 3 fg for a baculovirus particle. This observation was attributed to the fact that the resonance frequency and the associated shift increased with decreasing cantilever length. Instead of specific antibodies, Braun et al. used the interaction between the T5 bacteriophage and its prey, *E. coli*, for the detection of the viral particles [122]. Quantitative mass-binding measurements of T5 phage were performed at sub-pM concentrations with a noise level of ±0.5 ng, and the mass of a single T5 particle was measured to be 8 fg. A recent study by van den Hurk et al. detected bovine herpesvirus-1 (BHV-1) on a 10 nm-thick AlMo membrane of the resonator. The accumulated mass of BHV-1 varied depending on the membrane coating, with 7 ± 1 ng collecting on an active monoclonal 3D9S antibody-coating and 3.1 ± 0.1 ng on the control antihuman INF-γ antibody-coating [76]. 

The representative molecular and subcellular target samples discussed above are summarized in Table 2, and are listed according to the measured parameters and the stated purpose of the demonstration. Information on the type of device used for each target sample and the measurement conditions is also included.

#### 4.1.2. Applications and Perspectives 

Beyond proof-of-concept demonstrations, resonant MEMS technologies have also been used to answer biological questions that cannot be addressed through conventional means such as characterizing the effect of ionizing radiation on single DNA molecules. X-ray irradiation is commonly used in cancer radiotherapy, though our knowledge of the mechanisms by which the radiation kills tumour cells is based primarily on empirical observations of the overall cellular response to DNA damage. By using microgrippers, Perret et al. [88] performed a detailed biomechanical characterisation of DNA bundles exposed to X-ray radiation delivered by a therapeutic linear particle accelerator (LINAC). The DNA bundle degradation was detected as a reduction of bundle stiffness. Such characterisations, complemented with conventional cytological tests, may enable optimised radiotherapy solutions by providing a more detailed understanding of the mechanism behind radiative DNA damage and its effects on cellular populations. Furthermore, the same setup can be used to monitor DNA repair processes by mimicking cell conditions or using cell lysates. A similar technique was used to provide a chromatin analogue and monitor its mechanical properties to test epigenetic effects of *para*-sulphonato-calix[4]arene [90]. With this platform, DNA can be used for drug testing or combined therapy tests (radiotherapy and chemotherapy).

Another interesting application area for resonant MEMS devices is their use as toxin detectors due to their high sensitivity. As demonstrated by Ferrante et al., a microcantilever array as a biosensor system can detect aflatoxins in naturally contaminated nuts at concentrations of 40 pg mL^−1^ [110]. These biosensor systems can be extended to larger arrays, capable of measuring the interactions between many affinity ligands, and can be used for proteomics, diagnostics, or high-throughput screening applications.

Many of the presented devices can be used for diagnostic purposes as demonstrated for lung cancer [118], prostate cancer [73], and leukaemia [115]. Another potential diagnostic application demonstration was performed by Brunetti et al. With a sample volume of 6 µL, they could perform >50 experiments to detect multiple analytes simultaneously. The differential read-out with in situ controls minimises false-positive results. The functionality and practicality demonstrated by such devices shows their potential for routine diagnostic use in pandemic emergencies [117]. Further, coupled with downstream sorting modules, suspended micro and nanochannel resonators can be used for diagnostic purposes, for the detection of single nanoscopic particles, single macromolecules [69], or the real-time quantification of nanostructure assemblies [79]. Applications such as mass-based flow cytometry can be imagined with suspended channel resonators for the direct detection of pathogens or the non-optical sizing and density measurement of colloidal particles [11].

### 4.2. Working with Whole Cells

#### 4.2.1. Targets

In addition to subcellular targets, various types of silicon-based resonators have also been designed to work with cells of different natures: bacterial, fungal, and mammalian cells. In this section we introduce studies that have been performed to demonstrate the ability of resonators to characterize cellular samples.

*Bacterial cells* have been targeted by silicon-based resonators for over two decades. The surface chemistry of these devices can be easily modified, allowing the surface of a micro-cantilever to be coated with antibodies that specifically attach to target cells. One of the earliest demonstrations was by Ilic et al., [10] with the detection of *E. coli* O157:H7 cells. The number of cells attached on the surface of the cantilever was monitored through the shift in resonance frequency under ambient conditions (in air) and the authors showed that they could detect single cells. Using suspended channel resonators, Burg et al. managed to detect the mass of *E. coli* [11], which was followed by Godin et al. monitoring “instantaneous” growth using ultra-sensitive mass sensing [80]. A similar path was taken for analysing *B. subtilis*, which was first detected by Dhayal et al. [125], and followed by Burg and Godin for mass and growth studies [11,80].

MEMS-based systems can provide the femtogram sensitivity required for the detection of single cells. This degree of sensitivity can be of great use for the detection of blood stream infections such as sepsis; 33% of patients hospitalised with severe sepsis or septic shock die during treatment [126]. This high mortality is mostly due to the inability to rapidly detect and identify the relevant bacterial strains at early stages and administer the correct antibiotic regimen. Since biologically, a single cellular clone is considered to be able to start an infection, the ability to detect a single cell or as few cells as possible via changes in mass offers a straightforward opportunity to diagnose infections or diseases in a critically early timescale, and to monitor food or water supplies.

Besides enumerating and monitoring the growth of bacteria, MEMS devices can examine bacterial mechanisms such as osmoadaptation by observing the mechanosensitive (MS) channels [102]. Chang et al. measured the mechanical properties of a single *Synechocystis* cells. They compared the Young’s moduli of two groups: a group of wild-type cells and a group of genetically modified cells with a defect in the MS channels at three different osmotic concentrations to understand their physiological function in maintaining cell integrity.

*Fungal cells:* Performing growth measurements in humid air simplifies the cantilever functionalisation process by removing the need to attach the microorganisms on the sensor. In these measurements, the micro-cantilevers serve as miniaturised Petri dishes to detect the culture-based growth of any microorganism [127]. In conventional plating methods, the readout of microbial growth requires 24 h, whereas resonant micro-cantilever methods allow a measurement of the active growth in a couple of hours. The absorption of water on the functionalised cantilever surface due to colony growth results in a resonance frequency shift that can be detected. Hegner’s group reported several studies where they used resonant micro-cantilevers for label-free detection of fungal forms (*A. niger* and *S. cerevisiae*) [127,128]. They showed a detection sensitivity of ~200 *E. coli* cells, and the mass sensitivity for detecting fungal strains was 1.9 pg Hz^−1^. Another example of performing growth measurements in humid air, coupled with an automated fibre-optic-based readout technique was demonstrated by Maloney et al. [129] who monitored the growth of a filamentous fungus (*A. niger*) over 48 h with an initial growth detection time of 4 h.

Implementing detection in an aqueous environment with physiologically relevant pH, as introduced by Burg et al. [11] with a suspended microchannel resonator, brings an undeniable advantage over the strategies performed in humid air. Single nanoparticles, sub-monolayers of adsorbed proteins, and single bacterium were measured in water with sub-femtogram resolution. Alongside cellular mass, the measurement of cell density is also important for studying cellular processes, e.g., cell cycle, apoptosis, differentiation, or malignant transformation [81]. Bryan et al. monitored changes in cell density during the cell cycle and showed that cell density increases prior to bud formation by depositing *S. cerevisiae* cells in a suspended channel resonator [13].

Studies targeting the mechanical properties of fungal cells have been reported in recent years as well. *S. cerevisiae* and *S. pastorianus* are two types of fungal cells that have been examined using MEMS squeezers by measuring Young’s modulus [104], pre- and post-rupture stiffness [95], and stiffness in different solutions [98].

*Mammalian cells* have been the most commonly targeted cells to analyse with silicon-based resonators in recent years due to their potential impact on clinical studies. We can divide the primary measurement parameters as either physical, e.g., mass, or mechanical, e.g., stiffness. Similar to the previously explained cell types, physical parameters include mass [12], volume, and density [56]. These parameters allow monitoring some biological functions, e.g., cell growth [43,74,82], which leads to several applications such as testing drug response [40], sensitivity, and resistance [38]. Mechanical properties, on the other hand, include stiffness [130] and viscoelasticity [75].

Studies targeting physical parameters mainly use two designs: suspended plate resonators and cantilever-type suspended channel resonators. These devices have been mainly used to analyse cancer cells such as human colon cancer cell lines [12], breast cancer cell lines [43,63,74], human and mouse lung cancer cell lines [35,56], multiple myeloma cell lines [40], glioblastoma cell lines [38], mouse lymphoblastic leukaemia cell lines [35,36,38,41,56], and acute lymphoblastic leukaemia primary cells [38].

We must also note some demonstrations using suspended cantilever structures such as the work of Martinez-Martin et al. By measuring the total mass of adherent mammalian cells in culture conditions over days with millisecond time resolution and picogram mass sensitivity, they observed intrinsic mass fluctuations of around 1–4% over timescales of seconds throughout the cell cycle as a result of basic cellular processes including ATP synthesis and water transport [44]. The technique was applied to fibroblasts and HeLa cells (with and without vaccina virus infection) to study the link between mass fluctuations and cellular growth.

Most of these studies are based on the correlation between the resonant frequency shift and the change of effective mass of the cantilever due to cell attachment or change in its density through cell cycle. However, the characterisation of the mechanical properties of mammalian cells, e.g., their stiffness or viscosity, is also of great interest for cellular biology, tissue engineering, and especially in oncology [131]. Cells in our body are constantly exposed to mechanical stress, which plays a vital role in influencing many cellular processes such as the regulation of the cell cycle [132], apoptosis [133], cell growth [134], and migration [135,136]. Furthermore, these mechanical properties are often altered in diseased cells including circulating tumour cells (CTCs), for which deformability is considered to be an identifiable biomarker of malignancy [137].

**Figure 3:**
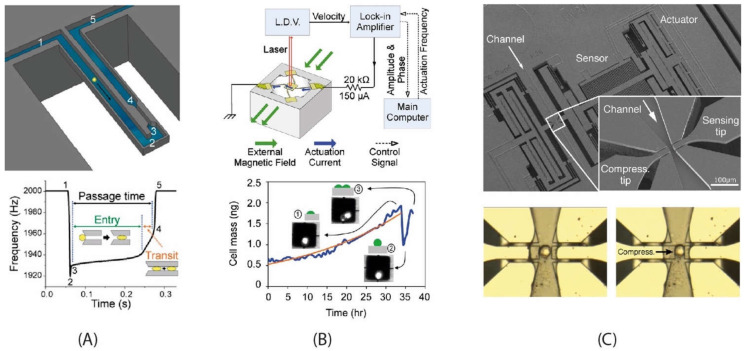
Examples of silicon-based resonating MEMS technologies for analysing cells. (**A**) The cantilever-type suspended channel device coupled a constriction located at the apex of the channel, described by Byun et al. [35] (with granted permission from PNAS). The cell (represented as the yellow sphere) is deformed by the 6 μm-wide, 15 μm-deep, and 50 μm-long constriction. The numbers 1 to 5 indicate the trajectory of the cell. The resonant frequency change of the cantilever structure changes with the cell passing in the channel and going through the constriction. (**B**) Suspended plate resonant sensor described by Park et al. [12] (with granted permission from PNAS), where the cells are cultured on a sensor platform and the increase in mass through cellular growth is measured. The graph on the right monitors a cell division event. Prior to cell division, an individual cell’s growth data (blue line) conforms to an exponential curve fitting. Insets 1–3 show the cell division event. (**C**) The fluidics-integrated MEMS squeezer device, described by Takayama et al. [65], has only the tips of the device enter the microchannel while the sensing and measurement components are not submerged, allowing simultaneous electrical and mechanical measurements in air (CC BY license).

Suspended plate resonators and cantilever-type suspended channel resonators have also been applied to analyse deformability [35] and viscoelasticity [75]. Byun et al. modified their suspended channel resonator design to added a constriction at the apex of the microchannel in the suspended resonator (Figure 3A) [35]. The cell’s buoyant mass, passage time, and velocity upon entering the constriction (transit velocity) was measured with a throughput of around one thousand cells per hour to quantify the cell stiffness. Corbin et al., on the other hand, used suspended plate resonators (Figure 3B) to extract the viscoelastic properties of single adherent cells by monitoring changes in the vibrational amplitude of their resonant sensor platform [75].

MEMS squeezers have also been used for mechanical characterisation of mammalian cells in recent years. Considering their geometry, the use of microgrippers is uniquely suited to the manipulation of individual cells. Baëtens et al. [130] inserted the tips of their microgrippers into a microfluidic channel via a side opening to capture individual breast cancer cells for characterizing their mechanical properties. The protruding tip geometry allowed access to single cells without compromising sensitivity by keeping the sensor and actuator working in air. Pekin et al. designed another approach to enter the channel via a top opening to allow integration with different imaging techniques such as confocal microscopy to perform mechanical characterisation of breast cancer cells during subcellular imaging [91].

Microgrippers can also be augmented with an internal microfluidic channel. The two opposing tips that compress and sense cells are positioned across from each other at the two sides of a channel. Cells of interest can be brought to the characterisation area via a flow driven by vibration [94] or microfluidic pumps [34,65]. While Sugiura et al. [34] measured the deformability of individual Madin–Darby Cannie Kidney (MDCK) cells with a fully immersed MEMS squeezer, Takayama et al. [65] designed a system that operated with an air–liquid interface (Figure 3C). Similar to the cantilever resonator design of Park et al. [112], the movable tips and the channel walls formed a small opening (4 µm) where the surface tension of the liquid prevented any leakage. As a result, the actuating and sensing elements of the MEMS device were kept in air working efficiently while the tips were immersed in liquid to compress and sense each cell [65].

A more recent study by Rezard et al. attempted to perform mechanical characterisations in a high-throughput format with a design change in the compression side. Instead of using a movable tip, Rezard et al. designed a wall that converged towards the sensing tip to form a gap smaller than the cell [97]. As a result, breast cancer cells in a continuous flow were compressed between the wall and the sensing tip while the stiffness of each passing cell was measured.

The cellular target samples introduced above are summarized in Table 3 according to the measured parameters and the analysis purpose. The device types used and measurement conditions are stated for each target sample.

#### 4.2.2. Applications and Perspectives

Using their suspended plate resonators, Corbin et al. demonstrated the difference in growth rates between highly invasive MDA-MB-231 breast cancer cells and noninvasive MCF-7 breast cancer cells and compared them with MCF-10A cells, showing that the benign cells have a higher mass growth rate than their cancerous counterparts [43]. A very similar system was used by Park et al. [12] to measure mass and growth rates of single adherent cells. Human colon epithelial cells were grown over the sensors for >50 h. The authors showed that the average growth rate increased linearly with the cell mass, at 3.25% per hour. The correlations made between cell mass and cell growth in this study are also relevant for the investigation of cell cycle progression. These techniques can be coupled with fluorescent imaging to follow fluorescent reporters and thus enable the study not only of the cell cycle, but also processes such as autophagy, apoptosis, and cell differentiation.

After detecting the mass of passing cells with high sensitivity, Manalis et al. made creative changes to the suspended channel resonators’ design and protocol to demonstrate several biological applications. They included density as another physical parameter to analyse cells using a dual resonator technique [56]. The culture media containing the cells passed through the first resonator for a buoyant mass measurement. Then, along the channel between resonators, a high-density fluid was introduced via a cross-junction and mixed with the cell culture media via diffusion in a serpentine channel. As the last step, the second buoyant mass measurement was recorded in the mixed fluid while cells were flowing through the second resonator. With this strategy, they showed that the mass and volume of the hematopoietic cell-line (L1210) were lower than those of the H1650 cells, which are of epithelial nature.

An important step towards practical applications was to demonstrate the ability to actively detect cell growth. Godin et al. used a bidirectional flow through the suspended channel: once a cell is detected, the flow direction is automatically reversed to reintroduce the cell back to the micro-cantilever [80]. Cermak et al. [39] used a series of resonators (10–12) along the channel with serpentine delay channels providing 4 to 20 min of on-chip incubation between each measurement. Cells in the suspension were flowed in a queue to achieve a throughput above 60 cells h^−1^ with a resolution of 0.2 pg h^−1^ for mammalian cells and 0.02 pg h^−1^ from bacteria. This system could identify subpopulations of cells with divergent growth kinetics and provides a great drug-testing platform with a significant advantage over conventional strategies that rely on bulk analysis. The difficulty in performing optical detection in a high-density array could be solved by fabricating the resonator array with piezoresistive sensors [139]. As a variation on cell growth analysis, Stevens et al. developed the single-cell mass accumulation rate (MAR) as a parameter to test the drug sensitivity and resistivity of glioblastoma and B-cell acute lymphocytic leukaemia cells [38]. They found not only a heterogeneity in drug sensitivity between these two types of tumours but also heterogeneities in drug response within the same tumour. Defined as the change in mass over time, MAR was determined through repeated weighing cycles (every ~30 s) over a ~15-min period. As the cell viability is preserved over the course of measurement, downstream genomic analysis is possible with these methods. Cetin et al. applied the MAR measurements to determine the therapeutic susceptibility of multiple myeloma to a given treatment [40]. According to the results, the MAR assay could correctly predict the response of nine patients to standard-of-care drugs according to their clinical diagnoses. This demonstration shows how suspended channel resonators can be used as tools for predicting therapeutic outcomes using clinical samples.

As discussed previously, Byun et al. added a constriction at the apex of the microchannel to compress passing cells. Using this technique to compare the transit velocity of different cell types, the cell lines with a higher metastatic capacity were shown to have shorter passage times than their low malignant counterparts [35]. This strategy, coupled with a downstream DNA sequencing step can be used for the analysis of CTCs to investigate the presence of genetic alterations that potentially modulate surface friction of cells and assess the relevance of these properties as possible drivers of metastasis. A suspended channel resonator design was also adapted to integrate with acoustic scattering by Kang et al. to monitor and analyse single cell mechanics [41]. This device used the size-normalised acoustic scattering (SNACS) method to measure stiffness through noncontact means.

The bridge-type suspended channel resonator, introduced by Martín-Pérez et al., combined mechanical mass measurements with optical reflectivity measurements [63]. Using a glass capillary bridge, a focused laser beam provided simultaneous resonance frequency and reflected optical power information as cells flowed past. The results demonstrated that mechano-optical techniques can successfully discriminate pathological (MCF-7 human breast adenocarcinoma cells) from healthy cells (MCF-10A nontumorigenic cells) of the same tissue type.

### 4.3. Working with Cellular Aggregates, Tissue, and Whole Organisms

#### 4.3.1. Targets

Spheroids are aggregates of cancer cell lines cultured in suspension or on scaffolds to mimic in vivo tumours. When compared to conventional monolayer (2D) cell-cultures, these 3D cultures are more accurate for testing tumour growth, hypoxia, and drug response [140,141]. During uncontrolled growth, a tumour inevitably applies pressure onto and receives compression from the surrounding tissue. These mechanical interactions may contribute to key developments in tumorigenesis [142] and are therefore important to assess. Sakuma et al. [14] described a robot-integrated microfluidic chip (robochip) to evaluate the stiffness of cell spheroids and used this method to evaluate the changes in stiffness through culture time. The robochip contained a microchannel and a pair of V-shaped on-chip probes with a force sensor. A piezoelectric actuator compressed the whole chip, which is deformed, and the relation between the deformation ratio and the spheroid reaction force was measured giving a stiffness index, which increased with culture time.

Tissue handling can be a very demanding task requiring both skill and expertise. One example of such a task is blood vessel dissection, wherein the microvessel needs to be manually dissected and fixed into an oxygen supplemented saline bath with tungsten wires. To improve the existing procedures in terms of accuracy, reliability, and ease of operation, Wierzbicki et al., described an electrostatically driven silicon microgripper with a tilt compensation mechanism for blood vessel manipulation and measurement of the contraction force of blood vessels [46].

A more recent study used a fluidics-integrated device to independently apply and sense both tensile and shear forces in an epithelial cell monolayer. Garcia et al. demonstrated that epithelia exhibit concomitant higher maximum resistive tensile forces and quicker force relaxation. Also, the maximum resistive forces of epithelia under cyclic shear perturbation remained unchanged between cycles, and cyclic loading led to faster relaxation of the resistive forces [45]. The use of this device can be extended for pharmacological perturbation of cell structures and functions.

#### 4.3.2. Applications and Perspectives

Applying stimulation and sensing mechanisms to larger organisms leads to different difficulties due to large sizes, displacement requirements, and high force levels when compared with molecular or single-cell approaches. Kawahara et al., sought to analyse an aquatic microorganism, *Pleurosira laevis* [47]. They developed a magnetically driven microrobot to overcome the difficulties caused by the size and geometry of the organism. The quantitative evaluation and analysis were based on magnetically driven stimulation and optics-based beam-deformation sensing.

More complex biological systems such as insects or worms are widely used as model organisms. The fruit fly *Drosophila melanogaster* is a common model system for cellular, genetic, and developmental processes. On the other hand, the fruit fly also possesses a highly developed flight control mechanism that can be a source of design inspiration for biomimetic engineering. However, obtaining accurate measurements of a 3 mm-long insect in-flight is challenging. For studying the flight dynamics of *Drosophila melanogaster*, Sun and coworkers used MEMS-based capacitive force sensors. Individual flies were tethered to the MEMS sensor probe by a tungsten wire glued to their thoraxes and real time measurements were performed for each wing stroke. The average lift force was measured as 9.3 µN, which is within the range of typical body weight for the fruit fly.

Such technologies can be expanded for the measurement of other small multicellular organisms for the assessment of their propulsion forces or the investigation of mechanisms behind their motility. This could have applications not only for biomimetic engineering purposes but also, in the case of parasites, in the elaboration of new therapeutic strategies.

## 5. Concluding Remarks

The striking label-free detection capability of MEMS resonators can achieve single-molecule resolution [16], which overcomes the “diagnostic grey zone” limitation of commonly used techniques such as the enzyme-linked immunosorbent assay (ELISA) [143] and makes them promising candidates for use in clinical diagnostic tests. Furthermore, over the years, MEMS resonators have evolved from relatively simple detection devices to become devices capable of intricate analysis of cellular functions for drug testing and other applications [38]. Besides physical properties (e.g., mass or volume), these devices can also measure mechanical (e.g., stiffness and viscosity) and biological properties (e.g., cell growth). Although earlier demonstrations could not match the high throughputs of microfluidic-based cytometry techniques such as deformability cytometry, recent developments have demonstrated the potential to analyse hundreds of cells per second with only electrical readouts and without relying on imaging for analysis [97,144].

Besides measuring different physical or mechanical parameters, MEMS resonators can also be integrated with other techniques to provide multi-parametric analysis at the molecular or single-cell levels such as the combination of hybrid surface plasmon resonance (SPR) and cantilever-based mechanical sensing platforms [119]. Recent examples include the measurement of cell mechanics by acoustic scattering in conjunction with biophysical properties obtained by a suspended microchannel resonator [41]. Similarly, impedance spectroscopy can be combined with mechanical characterisation at the single-cell level with a MEMS squeezer [97]. Demonstration of such hybrid sensing platforms can lead to diagnostic tools to discriminate cells using multi-parametric analysis.

Some of the techniques are already relatively mature, e.g., suspended channel resonators. Over the years, they have evolved from molecular/cellular detection devices to tools that can be used for clinical applications such as monitoring drug sensitivities. MEMS squeezers, on the other hand, have started attracting more attention in recent years as a result of the increasing interest in the mechanical properties of biological samples. Although microfluidics-based, high-throughput mechanical cell characterisation techniques [33] have been demonstrated successfully, design, fabrication, and automation possibilities still make resonating MEMS devices promising tools for routine clinical use.

To summarise, silicon-based resonating MEMS represent not a single product or a stand-alone strategy but rather a versatile engineering toolkit that can be used for diverse applications ranging from the analysis of single molecules to the biophysical characterisation of prokaryotic or eukaryotic cells and even to the study of more complex, multicellular organisms. Silicon-based, micro-/nanofabricated tools benefit from the long-established and very advanced protocols of the semiconductor industry with advantages including high throughput production and the absence of architectural randomness. Mechanical elements, sensors, and actuators can be all integrated on a single silicon substrate and the surface chemistry can be easily modified for affinity-based detection. Through the integration of microfluidic systems, conventional cell culture procedures, immunoassays, and tissue engineering strategies can be miniaturised to use lower sample volumes and be performed in a fewer number of steps. With their unprecedented sensitivities and integration possibilities with integrated circuits and other measurement techniques, MEMS resonators are moving towards practical medical applications as potential point-of-care devices.

In this review, we have focused on silicon-based resonating MEMS analysing biological samples; although, today, MEMS technologies in general are already widely used in our everyday life in the form of accelerometers and gyroscopic MEMS devices for smartphones and game controllers. Recently, in order to apply these fabrication processes to the biomedical field, development has begun to accelerate for applications in diagnostics, biosensors, or drug delivery due to the inherent advantages that MEMS can provide. Physical interactions can be miniaturised nearly to the same degree as integrated circuits, reducing sample volumes while simultaneously integrating sensing and analysis components. Over the following years, BioMEMS will provide a new vision of the medical sciences and change how we perceive biological entities.

## Figures and Tables

**Table 1:** An overview of the silicon-based resonator types.

Device Type	Sample	Parameters	Stimulation/ Sensing	Key Fabrication Steps	Ref.
**Suspended structures**					
Cantilever 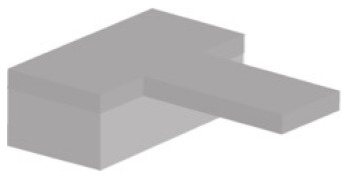	Molecules, Proteins, Nucleic acids, Viruses	Mass, Viscosity, Density	Thermal/Optical	- Etching (RIE, vapour) - Deposition (PECVD)	[8,107] [108]
			Piezoelectric (ext)/ Optical	- Etching (RIE, wet) - Deposition (LPCVD)	[109,110] [111]
			Piezoelectric/ Optical	- Deposition (PECVD) - Sacrificial layer - EB lithography - Etching (RIE)	[9]
			Optical/ Optical	- Etching (DRIE, wet)	[112,113]
				- Deposition (LPCVD) - Sacrificial layer - EB lithog., Lift-off - Etching (RIE)	[101]
			Electromagnetic/ Electromagnetic	- Lift-off - Etching (DRIE)	[114]
2. Bridge 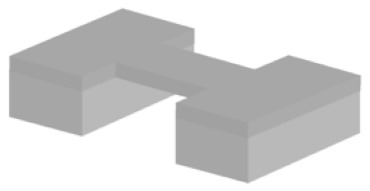	Proteins	Mass	Piezoelectric (ext)/ Optical	- Deposition (PECVD) - EB lithography - Etching (RIE, KOH)	[62,71,72]
3. Plate 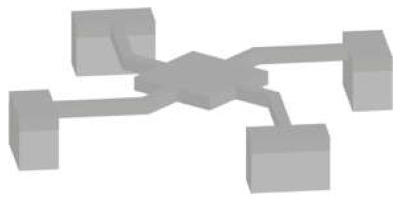	Proteins	Mass	Piezoelectric (ext)/ Optical	- Oxidation - Thin-film depo - Etching (RIE, HF)	[73]
	Cells	Cell mass, Cell growth, Stiffness, Viscoelasticity	Magnetic/ Optical	- Deposition (PECVD, Au) - Etching (Vapour XeF_2_)	[12,42,43,74,75]
**Suspended channel devices**					
4. Cantilever 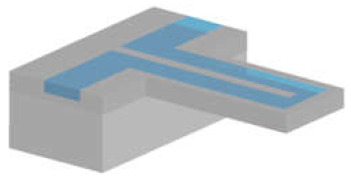	Proteins, Nucleic acids, Exosomes, Cells	Mass, Cell density, Cell volume, Cell growth, Deformability, Mass accum. rate	Electrostatic/ Optical	- Deposition (LPCVD) - Etching (RIE) - Sacrificial layer	[55]
			Piezoceramic (ext)/ Optical	- Wafer bonding (Si-Si, Si-pyrex) - Etching (RIE)	[11,13,35,38,56,80,81,82,115]
					[79]
			Electrostatic/ Piezoresist	- Ion implantationDeposition (PECVD)	[70]
			Piezoceramic (ext)/ Piezoresist		[39,40]
5. Bridge 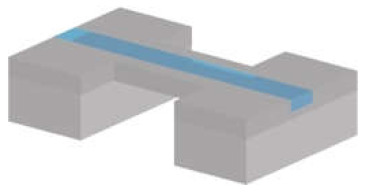	Cells	Mass	Optical/ Optical	- Sacrificial layer - Etching (DRIE, wet) - Polymer coating (parylene)	[63,83,84]
6. Plate 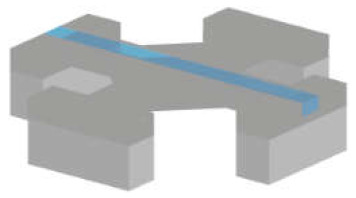	Buffers, Solutions	Mass	Electrostatic/ Electrostatic	- Etching (DRIE) - Wafer bonding - Oxidation	[64,85]
**MEMS squeezers**					
7. Microgrippers 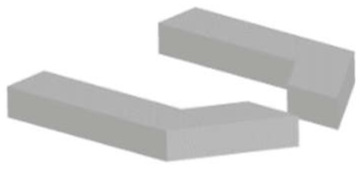	DNA, Cells, Animals	Force, Stiffness, Young’s modulus, Viscosity, Elastic modulus,	Electrostatic/ Capacitive	- Etching (DRIE, wet) - Deposition (LPCVD)	[15,48,57,58,88,89,90,91]
	Drug capsules		Electrothermal/ Capacitive		[116]
8. Fluidic integrated device 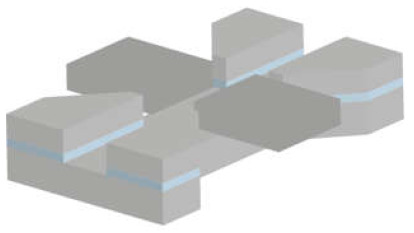	Proteins, Cells, Cell spheroids, Microorganism	Force, Stiffness, Young’s modulus, Viscosity, Elastic modulus	Piezoactuator (ext)/Optical	- Etching (DRIE) - Glass-Si bonding	[14,34,94,102,104]
			Electrostatic or Electrothermal/ Optical	- Sacrificial layer - Bulk micromachining	[95,98,99]
			Electrostatic/ Capacitive	- Etching (DRIE, wet)	[65,97]
			Electrostatic/ Optical	- Deposition (LPCVD) - Sacrificial layer - Etching (DRIE, wet)	[96,100]
			Electromagnetic/ Optical	- Etching (DRIE) - Electroplating (Ni)	[47]

**Table 2:** An overview of the targeted molecular and subcellular biological samples. The device type column corresponds to the order of the devices introduced in Section 3.1 and Table 1.

Target Sample	Parameter	Purpose	Device Type	Condition: Sample/Measure	Ref.
**Molecules and proteins**					
Aflatoxins	Mass	Detection	1	Vacuum/Vacuum	[109,110]
Ochratoxin A					[109]
ALCAM	Mass	Cancer biomarker detection	4	Liquid/Vacuum	[115]
Tetrapeptide	Mass	Detection of proteolysis	1	Liquid/Liquid	[123]
Fibrinogen	Mass	Cancer biomarker detection	1	Air/Air	[111]
Collagen fibres	Stress, strain	Tensile mechanical resistance	8	Humid/Humid	[96,100]
Antigen, antibodies, *(IgG, biotin, avidin, EP9, SP3-E6, etc.)*	Mass	Surface coating	1	Air/Air	[107]
		Detection	4	Liquid/Vacuum	[55]
		Detection	1	Liquid/(partially) air	[112]
		Detecting binding rate		Liquid/Liquid	[124]
		Detection	2	Vacuum/Vacuum	[62]
		Testing malaria vaccine	1	Liquid/Liquid	[117]
PSA	Mass	Cancer biomarker detection	3	Vacuum/Vacuum	[73]
Insulin	Mass	Detection	1	Liquid/(partially) air	[113]
SOD1		Proteinase K enzyme reaction			
Matrix metallo-proteinase	Mass	Cancer diagnosis	1	Liquid/Liquid	[118]
**Nucleic acids**					
miRNA	Mass	Detection for cancer and liver injury diagnostics	1	Liquid/Liquid	[121]
ssDNA	Mass	Detection	1	Air/Air	[17]
		Enumeration		Vacuum/Vacuum	[101]
		Hybridisation kinetics		Liquid/Liquid	[61]
		Detecting hybridisation			[119]
DNA 110 bp,10 kbp	Viscosity, Density	Rheological characterisation	1	Liquid/Liquid	[114]
DNA λ-phage	Stiffness	Effects of irradiation Effect of ions Effect of compounds	7	Liquid/Air	[88][57,58,89][58,90]
DNA 3776 bp	Mass	Enzymatic reaction monitoring	1	Liquid/Liquid	[120]
**Viruses and exosomes**					
Baculovirus	Mass	Single virus detection	1	Vacuum/Vacuum	[9]
Vaccinia virus	Mass	Single virus detection	1	Air/Air	[8,108]
T5 virus	Mass	Detection	1	Humid/Humid	[122]
Bovine Herpesvirus1	Mass	Detection	3	Vacuum/Vacuum	[76]
Exosomes	Mass	Mass distribution	4	Liquid/Vacuum	[79]

**Table 3:** An overview of the targeted cellular biological samples. The device type column corresponds to the order of the devices introduced in Section 3.1 and Table 1.

Target Sample	Parameter	Purpose	Device Type	Condition: Sample/Measure	Ref.
**Bacterial and parasite cells**					
*E. coli*	Mass	Detection	1	Air/Air	[10,138]
	Mass	Detection	4	Liquid/Vacuum	[11]
	Cell growth	Instantaneous growth	4	Liquid/Vacuum	[80]
*B. subtilis*	Mass	Detection	1	Liquid/Liquid	[125]
	Mass	Detection	4	Liquid/Vacuum	[11]
	Cell growth	Instantaneous growth	4	Liquid/Vacuum	[80]
*Synechocystis* sp. strain PCC6803	Young’s modulus	Osmoadaptation mechanism of cell membrane	8	Liquid/Liquid	[102]
*P. falciparum*	Density	Drug treatment	4	Liquid/Vacuum	[81]
**Fungal cells**					
*S. cerevisiae*	Cell growth	Fast growth detection	1	Humid/Humid	[128]
	Mass, density, vol.	Growth during cell cycle	4	Liquid/Vacuum	[13]
	Mass	Budding yeast cells	4	Liquid/Vacuum	[70]
	Cell growth	Detecting growth rate	4	Liquid/Vacuum	[80]
	Mass	Combined optical observation	5	Liquid/Air	[84]
	Stiffness	Discriminating viable cells	8	Liquid/Liquid	[98]
	Force	Cell rupture analysis	8	Liquid/Liquid	[95]
	Young’s modulus	Force-deformation curve	8	Liquid/Liquid	[104]
*A. niger*	Cell growth	Fast growth detection	1	Humid/Humid	[127,128,129]
*S. pastorianus*	Stiffness	Rehydration effect on mechanical properties	8	Liquid/Liquid	[99]
**Mammalian cells**					
Colon cancer cell lines (human) *HT-29*	Mass, growth	Adherent cell growth	3	Liquid/Liquid	[12]
	Viscoelasticity	Cell discrimination by mechanical properties	3	Liquid/Liquid	[75]
Breast cancer cell lines (human) *MCF7, MCF10A, MDA-MB-231, SUM159-PT*	Mass	Long-term growth meas.	3	Liquid/Liquid	[42]
	Mass, growth	Discriminating pathological cells	3	Liquid/Liquid	[43]
	Mass + reflectivity	Discriminating pathological cells	5	Liquid/Air	[63]
	Stiffness	Discriminating cells	1	Liquid/Liquid	[74]
			7	Liquid/Air	[130]
Lung cancer cell lines (human, mouse) *H1650, H1975, HCC827, T_met_ …*	Mass, density	Comparing physical properties	4	Liquid/Vacuum	[56]
	Deformability	Comparing metastatic potential	4	Liquid/Vacuum	[35]
Multiple myeloma cell lines	Mass accumulation Rate (MAR)	Detecting drug sensitivity and predicting therapeutic response	4	Liquid/Vacuum	[40]
Glioblastoma cell lines *U87, BT145, BT159…*	Mass accumulation Rate (MAR)	Defining drug sensitivity or resistance	4	Liquid/Vacuum	[38]
Lymphoblastic leukaemia cell lines (mouse) *L1210*	Deformability	Comparing metastatic potential	4	Liquid/Vacuum	[35]
	Mass, density	Comparing physical properties	4	Liquid/Vacuum	[56]
	Mass accumulation Rate (MAR)	Defining drug sensitivity or resistance	4	Liquid/Vacuum	[38]
	Mass + SNACS	Single cell mechanics	4	Liquid/Vacuum	[41]
	Growth rate	Drug response	4	Liquid/Vacuum	[39]
	Mass	Growth efficiency monitoring	4	Liquid/Vacuum	[36]
B cell acute lymphoblastic leukaemia primary cells	Mass accumulation Rate (MAR)	Defining drug sensitivity or resistance	4	Liquid/Vacuum	[38]
HeLa	Mass, growth	Fast mass fluctuations	1	Liquid/Liquid	[44]
Fibroblast (mouse)	Mass, growth	Fast mass fluctuations	1	Liquid/Liquid	[44]
	Deformability	Mechanical characteristics	4	Liquid/Vacuum	[35]
MDCK cells	Force	Mechanical characteristics	8	Liquid/Liquid	[34]
